# Lipomatous hypertrophy of the interatrial septum

**DOI:** 10.1590/0100-3984.2016.0165

**Published:** 2018

**Authors:** Renato Niemeyer de Freitas Ribeiro, Bruno Niemeyer de Freitas Ribeiro, Wolney de Andrade Martins, Lívia de Oliveira Antunes, Edson Marchiori

**Affiliations:** 1 Universidade Federal Fluminense (UFF), Niterói, RJ, Brazil; 2 Instituto Estadual do Cérebro Paulo Niemeyer, Rio de Janeiro, RJ, Brazil; 3 Hospital Casa de Portugal/3D Diagnóstico por Imagem, Rio de Janeiro, RJ, Brazil; 4 Universidade Federal do Rio de Janeiro (UFRJ), Rio de Janeiro, RJ, Brazil

**Figure 1:**
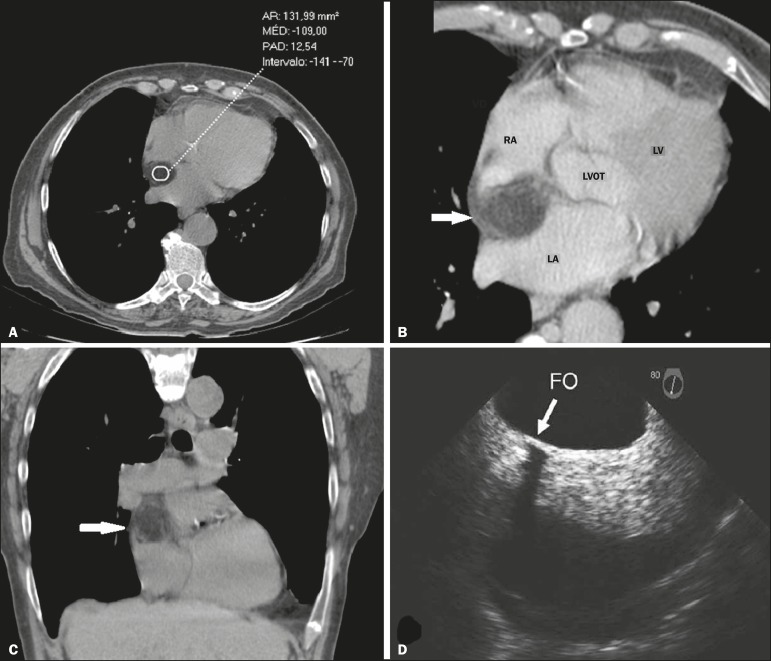
** A:** Non-contrast-enhanced CT scans showing IAS thickening of 2.4 cm with a density of −109 HU, characteristic of fatty infiltration. **B:** Contrast-enhanced CT with angled reformatting for the four heart chambers, showing thickening of the IAS with no evidence of contrast enhancement (arrow). **C:** Non-contrast-enhanced coronal CT scan showing fatty infiltration of the IAS (arrow). **D:** Transesophageal echocardiogram showing thickening of the IAS, sparing the fossa ovalis (arrow). RA, right atrium; LA, left atrium; LV, left ventricle; LVOT, left ventricular outflow tract; FO, fossa ovalis.

Dear Editor,

A 74-year-old female patient underwent screening for neoplasia due to weight loss in the last six months, presenting with no other complaints. She had hypertension and diabetes mellitus, both of which were well controlled with medication. During the investigation, computed tomography (CT) of the chest showed interatrial septum (IAS) thickening of 2.4 cm, caused by fatty infiltration, sparing the fossa ovalis (Figures 1A, 1B, and 1C). Complementary evaluation by transesophageal echocardiogram (Figure 1D) corroborated the previous findings. On the basis of those data, a diagnosis of lipomatous hypertrophy of the interatrial septum (LHIAS) was confirmed.

The evaluation of the cardiovascular system by imaging methods has been the objective of a series of recent publications in the radiology literature of Brazil^(1-4)^. LHIAS is characterized by excessive fat deposition in the IAS, sparing the fossa ovalis and expanding the transverse diameter of the IAS to > 2 cm^(5-9)^. The condition is more common among women and the elderly; it has also been associated with corticosteroid use, obesity, and pulmonary emphysema^(5-8)^. In most cases, LHIAS presents as an incidental finding on imaging examinations. However, in rare cases, it can be the cause of obstruction of the vena cava and cardiac arrhythmias, especially those of atrial origin.

Among the imaging methods employed in the evaluation of patients with suspected LHIAS, echocardiography shows a limited capacity for characterizing the tissue that composes cardiac masses, CT and magnetic resonance imaging (MRI) therefore being fundamental for further evaluation. Those methods are capable of identifying IAS thickening > 2 cm sparing the fossa ovalis, with or without a dumbbell-like morphology, as well as characterizing the fatty infiltration of IAS, defined as densities between −80 HU and −120 HU on CT and as a hyperintense signal in T1-weighted sequences, as well as a signal drop in fatsuppressed sequences, on MRI^(5,6,8,10)^.

Recent studies have highlighted the use of 18F-fluorodeoxyglucose positron emission tomography/computed tomography in the evaluation of LHIAS, showing that, for individuals with LHIAS, ^18^F-fluorodeoxyglucose uptake is greater in the brown fat deposited in the IAS than in the subcutaneous fat of the chest wall, because the former is metabolically active. That could represent an imaging pitfall, leading to an incorrect diagnosis of infectious, inflammatory or neoplastic lesion. To avoid misinterpretations, it is necessary to make the correlation with the CT and MRI findings^(6,7,11)^.

Because LHIAS is a benign condition, most cases do not require treatment, although surgery can be indicated in the rare cases in which there are symptoms secondary to the compression of structures, such as the vena cava and the pulmonary veins^(6,8)^. The main differential diagnosis is cardiac lipoma, which is encapsulated and affects the fossa ovalis. Another major differential diagnosis is liposarcoma, which is distinguished by atypia and by its rapid, aggressive evolution.

In conclusion, a diagnosis of LHIAS should be considered when there is > 2 cm of thickening, due to fatty infiltration, of the IAS, sparing the fossa ovalis. It should also be borne in mind that a diagnosis of LHIAS is more common in elderly patients.
